# Cognitive Frame and Time Pressure as Moderators Of Clinical Reasoning: A Case Control Study

**DOI:** 10.5811/westjem.24851

**Published:** 2025-07-11

**Authors:** Andrew J. Monick, Xiao Chi Zhang

**Affiliations:** * Sidney Kimmel Medical College at Thomas Jefferson University, Department of Emergency Medicine, Philadelphia, Pennsylvania; † Sidney Kimmel Medical College at Thomas Jefferson University, Department of Emergency Medicine, Philadelphia, Pennsylvania

## Abstract

**Introduction:**

Emergency physicians (EP) are uniquely positioned to benefit from a deeper understanding of cognitive bias, particularly in the context of limited processing time. The framing effect—the tendency to evaluate identical information inconsistently given varying methods of presentation— presents a particular challenge within emergency medicine (EM). Understanding how the presentation of clinical information affects medical decision-making is paramount, given variability in how information is received. In this study we aimed to assess whether the imposition of a cognitive frame and time pressure affected participants’ differential diagnoses.

**Methods:**

We recruited attending physicians in emergency medicine (EM) and third-year EM residents via email from our university hospital. They were asked to review two case vignettes: one consistent with pulmonary embolism (PE), the other with interstitial lung disease. Each vignette had two versions, one emphasizing features consistent with the respective diagnoses. Each pair of vignettes contained objectively identical clinical information. Subjects were randomly assigned to one of four conditions based on 1) the specific or non-specific-frame version of each case and 2) the inclusion or exclusion of time pressure. Subjects provided their top three differential diagnoses for each case. Our primary outcome measure was identification of intended diagnosis.

**Results:**

A total of 39 subjects completed the study. Two-sided Fisher exact tests showed that varying cognitive frames affected the likelihood of EPs identifying PE as a diagnosis of interest (*P* = .01). Among EPs who identified PE, the likelihood of this diagnosis leading their differential diagnosis was also related to frame (*P* = .01).

**Conclusion:**

The results of this work reveal that cognitive frame and time pressure may independently influence diagnostic reasoning among emergency physicians, bearing implications for medical education.

## INTRODUCTION

Diagnostic errors are a major source of preventable harm in medical care. Between 40,000–80,000 deaths result from misdiagnosis in the United States each year,^1^ and approximately 5% of autopsies reveal a diagnostic error that, if identified during diagnosis, could have prevented the patient’s death.^2^ As of 2013, diagnostic error was the leading category of medical malpractice claims and accounted for the highest proportion of total payments.^3^ As medicine advances, more viable therapeutic options become available when a condition is accurately diagnosed. A delay in implementing these options for management means that diagnostic error is more likely to allow for progression to an intractable stage of disease that might otherwise have been averted.^4^

A key and under-investigated source of diagnostic error is the framing effect. The framing effect is a type of cognitive bias that manifests as the tendency to evaluate identical information inconsistently given varying presentation methods.^5^ For instance, patients who are told that a procedure has a 5% mortality rate may be less likely to elect for a procedure than those who are told that 95% survive. This phenomenon presents a particular challenge to clinicians and significantly impacts patient care.^6^–^9^ Although factors that exacerbate the framing effect in medical care are less comprehensively understood, reduced processing time may also negatively affect physician decision-making.^10^

Physicians may be especially susceptible to cognitive bias in the acute care setting,^11^,^12^ possibly contributing to the high diagnostic failure rate in fast-paced, information-limited clinical specialties such as emergency medicine (EM). Previous literature suggests that diagnostic failure in EM ranges from 10–15% overall^13^ and can reach up to 55% when assessing more elusive pathologies.^14^

Emergency physicians (EP) are expected to interview, examine, diagnose, and manage patients with a diverse spectrum of disease, while lacking the benefits of trust and history afforded to an established primary care physician. The acute nature of emergency department (ED) complaints means that patients may also not be able to provide accurate histories of present illness given varying levels of consciousness, increasing reliance on information filtered through the lens of other healthcare professionals.^15^,^16^ The unpredictable and chaotic environment inherent to the typical ED makes these challenges still more difficult. Clinicians face pressure to integrate limited and ambiguous data to make simultaneous and rapid medical decisions about undifferentiated patients, and a single mistake can lead to downstream patient morbidity and mortality.^16^,^17^

A key source of cognitive bias is the misuse of heuristics—cognitive shortcuts. Although heuristics carry inherent limitations, they are necessary to efficiently practice EM.^13^,^18^ As a result, the corresponding need for EPs to self-monitor for cognitive bias is paramount. Moreover, understanding the effect of time pressure on diagnostic accuracy is vital within medical specialties such as EM in which EPs must balance multiple patients simultaneously, and a delay in treatment could dictate life or death.^19^

While previous studies have assessed the isolated effects of the framing bias and time pressure on diagnostic reasoning, how the confluence of the two affects clinical acumen has not been previously uninvestigated. In this study, we evaluated these two factors in tandem to model the likely experience of an EP in daily practice. We asked EPs to evaluate hypothetical case vignettes and provide their top three differential diagnoses to investigate whether cognitive frame-attributed changes in differential diagnoses generated by EPs were exacerbated by limiting processing time.

## METHODS

### Study Design and Setting

This cross-sectional study was distributed electronically via an institutional Qualtrics account (Qualtrics International Inc, Provo, UT). We collected data over a two-month period between October–December 2021. The Thomas Jefferson University Institutional Review Board (IRB) approved the study (approval #21G.084). All experiments were performed in accordance with relevant guidelines and regulations (such as the Declaration of Helsinki). Informed consent was obtained from all subjects.

**Population Health Research Capsule**

What do we already know about this issue?

*Diagnostic error affects patient safety. Cognitive bias is a key source of error in specialties like emergency medicine in which time and information are limited*.

What was the research question?

*Does the imposition of a cognitive frame and time pressure affect participants’ differential diagnoses?*

What was the major finding of the study?

*Varying cognitive frame, but not time pressure, affected the likelihood of emergency physicians identifying a diagnosis of interest (P = .01)*.

How does this improve population health?

*Understanding factors that affect diagnostic reasoning is especially important when managing vulnerable patients for whom costly testing is not accessible*.

Participants were recruited using an email listserv of EPs at Thomas Jefferson University in the metropolitan Philadelphia, PA, area. Inclusion criteria included attending EPs and third-year EM residents who were employed at a hospital affiliated with the Jefferson Health System. Demographic information collected, including age range, sex, and years of post-residency practice, was not identifiable. Participants were enrolled via email and invited to complete the study at their convenience. A $20 prepaid debit card was offered to participants.

### Interventions

To assess for cognitive bias, we used two cases from a New Zealand study related to cognitive bias by Popovich et al.^9^ These cases, originally written to address complaints germane to emergency and internal medicine, were selected with the goal of replicating findings among a novel cohort of EPs while minimizing confounders. While interstitial lung disease (ILD) is not a commonly diagnosed pathology in American EDs, we incorporated both cases with the goal of more broadly reproducing the authors’ findings. We included a unique variable through the imposition of time pressure. Each case has two associated vignettes (four vignettes total), which we modified slightly to reflect American medical terminology and US conventional references, ranges, and units. After reading each vignette, participants were asked to provide their top three differential diagnoses.

The pair of vignettes associated with each case (Appendices 1 and 2) contain identical objective clinical information; the syntax varies, but each can be rearranged to reproduce the other. The difference between each pair of vignettes lies in whether the information emphasized features consistent with a specific diagnosis, namely, pulmonary embolism (PE) or ILD. Cases that emphasized features consistent with specific diagnoses like PE would highlight familiar clinical correlates (ie, buzzwords) that classically cue this diagnosis, such as recent surgery, “hemoptysis,” “tachycardia”) at the beginning of the case. In contrast, a control PE case would scatter the same findings throughout the vignette, with non-specific terminology, such as “blood-tinged sputum,” “heart rate of 110 bpm,” and “past surgical history of cholecystectomy.” These differences constitute the operationalization of a framing effect.

To establish a feasible constraint for time pressure, we conducted a pilot study in which 11 EPs (excluded from the final study measurements) read the vignettes and provided their three most likely differential diagnoses. The time taken by each EP to read a vignette was measured as the difference between the time at which the text loaded and the time at which the EP advanced to the screen for diagnosis entry. Based on a review of the literature surrounding time pressure conditions for reading tasks,^20^,^21^ participants in the time pressure arm were allocated 62 seconds via a countdown timer displayed at the bottom of the survey screen. The survey automatically advanced to the entry of differential when this time expired.

The figure shows the process flow chart associated with this study. For each of the two cases, participants were randomly assigned to one of four groups in Qualtrics based on 1) a specific-frame or nonspecific-frame vignette and 2) the inclusion or exclusion of time pressure. This led to a 2×2 design. We intentionally presented the case associated with PE first to all participants as we hypothesized that it would have greater relevance to EPs given the disease’s life-threatening nature, thereby avoiding practice effects.

### Outcomes and Analysis

The primary outcome measure associated with each condition was the frequency with which EPs identified an expected diagnosis (PE for the first case; ILD for the second). The secondary outcome measure was the rank order for each recorded differential diagnosis. Two researchers (AM, XCZ) evaluated responses and recorded whether the disease suggested by the vignette appeared in the list of three diagnoses provided by each participant. Disagreements were resolved through consensus by study investigators. A diagnosis was scored based on whether it included the main component of the diagnosis or a commonly accepted abbreviation (eg, “PE” for pulmonary embolism). Disagreements were resolved through discussion. Using IBM Statistical Package for the Social Sciences (IBM Corp., Armonk, NY), we performed two-sided Fisher exact tests and unpaired *t*-tests to compare the groups.

## RESULTS

### Characteristics of Study Subjects

The study population included all EM attendings and third-year EM residents employed by Jefferson Health, comprising approximately 120 physicians. In total, 48 physicians (40%) responded to our request, 39 of whom completed at least one case and 36 who completed both. Participants were permitted to exit the study at any time. Missing data were handled by omission from further analysis.

A total of 37 participants provided demographic information. The modal age ranges were 26–30 (11) and 31–35 (11). Twenty-four respondents (64.8%) identified as male and 13 (35.2%) as female; no respondents endorsed a non-binary gender identity. Twenty-seven respondents primarily identified their ethnicity as White (73.0%) or Asian (7, 18.9%). One participant self-identified as Asian and White; another did not identify with a category of ethnicity. Four respondents were residents (postgraduate year 3), and 33 were attending physicians. The range of post-residency years of practice was 0–35, with a mean (SD) of 7.4 (8.5).

### Main Results

Across the two associated vignettes, only 9% of EPs identified ILD as a diagnosis of interest; accordingly, there were insufficient relevant responses from which to draw statistical conclusions. Beyond their nationality, it is unclear how our physicians differed from those who participated in the study conducted by Popovich et al,^9^ who identified ILD at a rate of 52%, particularly since rates of identifying PE in the corresponding vignettes between our two studies were closer (82% in our study vs 78% in Popovich et al) Accordingly, we conducted our analysis of frame using data gathered from the PE vignette (39).

Two-sided Fisher exact tests (FET) showed that varying cognitive frames affected the likelihood of EPs identifying PE as the intended diagnosis in the corresponding vignettes (*P* = .01, FET). We did not observe an association between the application of time pressure and the appearance of expected pathology in the differential diagnosis (*P* = .09, FET). However, there was a significant difference between the time EPs took to generate a differential diagnosis when time pressure was imposed. A two-sample *t*-test revealed that those participants exposed to time pressure used significantly less time (*t*_36_ = 3.091, *P* <.01).

We conducted a subgroup analysis among 32 participants who identified PE as a diagnosis of interest. In this group, PE was more likely to be first on a part icipant’s differential if exposed to a vignette with a specific frame (*P* = .01, odds ratio [OR]13.6, 95% confidence interval [CI] 2.2–85.9, FET). Similarly, PE was significantly more likely to be listed as the first diagnosis of interest by participants exposed to time pressure than by those who were not (*P* = .02, OR 13.0, 95% CI 1.4–121.4, FET). These results are summarized in Table.

We did not observe a significant relationship between the likelihood of identifying PE/ILD and age range or years of practice. Analysis when stratified by the presence or absence of time pressure was also unremarkable.

## DISCUSSION

Our goal in this study was to investigate the effects of cognitive frame and time pressure on diagnostic reasoning. We hypothesized that the imposition of frame and time pressure would lead to observable changes in clinical reasoning. Based on the variation in differential diagnoses observed among EPs who assessed our case suggestive of PE, it seems that cognitive frame affects diagnostic reasoning. We further discovered that imposition of a cognitive frame may influence the order in which EPs noted an intended pathology in their differential diagnosis. We did not observe a relationship between time pressure and diagnoses provided.

Our results affirm the findings of Popovich et al that varied cognitive frames lead to significant differences in the generation of differential diagnoses. Emergency physicians were more likely to list PE as their first-line diagnosis when a frame toward PE was imposed. Although typically investigations are ordered to rule out any life-threatening pathologies in the ED, this finding may be most relevant in cases where time is most limited. When an EP must make a decision to initiate treatment quickly, accurately identifying the primary diagnosis of interest is critical. If reasoning can be swayed by frame and time pressure, it warrants considering whether a specific analytical approach might be consciously implemented in cases of the highest acuity.

We did not find a statistically significant relationship between time pressure and EPs’ likelihood of identifying an expected diagnosis in their differential. One might expect EPs to default to heuristics and select a more easily retrieved diagnosis given the imposition of time pressure, and prior research has corroborated the logical suspicion that time pressure leads to impaired diagnostic reasoning.^10^ However, other work showed that the two variables are independent. Norman et al^22^ and Monteiro et al^23^ each found that no differences in diagnostic accuracy emerged between groups who were asked to assess a case as fast or as thoroughly as possible, respectively. The variation among findings may be attributed to variations in experimental design, including the difficulty of the simulated cases and the intensity of time pressure imposed, and the experience levels and specialty training of participants.

At the core of this study is the principle that a clinician may arrive at a different conclusion if objective data are arranged or delivered differently. Emergency physicians commonly leverage external record-reviewing as a source of composite information about a patient’s medical history, as they may not be able to recall or relay specific complexities of prior care. Diagnoses ascribed in prior visits provided rationale behind ambulatory referrals, and choices of narration in a hospital course are both crucial for understanding of patient care and influential in how a patient will be managed. Awareness of cognitive bias is crucial for physicians whose jobs are intractably tied to time constraints and limited information. Future studies might assess the role of cognitive bias based on whether physicians prioritize narrative text from others (eg, discharge summaries) or objective, documented data (eg, lab results) when receiving a patient.

Differential diagnosis is especially important in EM, where undifferentiated patients present for an initial point of care; moreover, the patients who receive primary care in the ED are often those who are the most vulnerable.^24^,^25^ A broad differential diagnosis is critical for disposition planning and outcomes. Interestingly, an EP’s differential can prime those physicians who later assume the patient’s care, resulting in further unwarranted diagnostic momentum.^26^

The clinical paradigm affected by the framing bias includes a physician’s cognitive services—what doctors can offer when diagnostic tests are not viable, whether for time, expense, or clinic resources.^27^ As the number of uninsured individuals in the United States continues to increase,^28^ a physician’s capacity to diagnose without the use of costly studies remains critical. The expanding population of patients who stand to benefit the most from this research may be those who require immediate care and cannot afford comprehensive care.

Moreover, patients increasingly wish to be a part of decisions made about their care, a process known as shared decision-making.^29^,^30^ Perceived misdiagnosis was found to be the most commonly reported complaint levied against EPs.^31^ The results of this study affirm that it is incumbent upon the physician to not only successfully de-bias patient data while evaluating a case but also to present information in a way that does not impose a cognitive frame for the patient.

We would be curious to see how this effect bears out in a simulated session, in which information is presented verbally, as this would more accurately represent how information is processed in the clinical setting. If borne out in research, a logical next step from this work would be to explore how to mitigate diagnostic errors attributable to cognitive bias. Affirming evidence in this domain is somewhat limited. A recent review by Hartigan et al^32^ explores a variety of strategies but notes that evidence supporting error reduction is thus far weak. For instance, while there is a strong theoretical background for using cognitive-forcing strategies,^33^ they have, in practice, been shown to fail to reduce diagnostic errors.^34^,^35^ Deliberate, guided reflection seems to be the debiasing strategy with the strongest empirical promise thus far^36^,^37^; however, future study is needed.

## LIMITATIONS

The primary limitation of this study is its qualified verisimilitude to realistic decision-making processes in a clinical setting. In the ED, an EP may receive information verbally, through a narrative in text, as data in tabulated form, or, commonly, some combination of the three. Moreover, while patient information may be collected and refined in advance, as with our case vignettes, it may arrive in chunks, leaving the EP to process relevant data in real time. Susceptibility to frame may be weakened or exacerbated by this mutability of data presentation in the clinical setting.

This study presented data in text, which does not account for the complexities above and does not account for differing rates of reading or limitations such as dyslexia. Given the remote, asynchronous distribution of the survey instrument, neither were we able to discern whether other distractions were at hand and whether participants were allocating their full attention to the task, which might have affected both the differential diagnosis provided and time taken. A benefit of this approach is the removal of real-life confounders present in the typical ED, isolating the impact of the cognitive frame and time pressure over diagnostic reasoning. This is both a limitation with regard to clinical applicability and a strength with regard to studying the effects at hand.

Another limitation of this study is construct validity. While the core methodology of this paper reflects a previously published study to assess the framing effect, other cognitive biases and principles of reasoning are likely also at play. The availability heuristic, for instance, likely played a role in the ILD vignette, wherein the evaluating EP likely encountered a similarly presenting or mentally prominent case more recently. More broadly, the conditions of this study evoke thinking along the two prongs of dual-process theory, wherein System I is fast and intuitive and System 2 is slow and deliberate.^32^ Although the intent of this work was to place emphasis on adjusting for frame, future work might examine the interplay between these constructs. Nuanced simulation cases might be better able to distinguish how EPs respond to cases when each is invoked individually.

Over the course of this study, it occurred to us that a more natural fit to study the framing bias within emergency care may be during sign-out rather than during the initial patient presentation. Framing is a significant aspect of transferring care of a patient between EPs, during which the departing physician elaborates upon their dominant line of thinking to alert the incoming physician to key points in patient care. For instance, mitigation of frame in this context might include expanding one’s presentation to include a differential. We propose further study to investigate how sign-out culture can be assessed and reformed within the context of this cognitive bias.

We also encountered unexpected difficulty with the EP evaluation of the case vignettes suggestive of ILD. We could not evaluate for effects related to the ILD cases given the low frequency of identification within differential diagnoses. While nitrofurantoin-induced ILD is a rare occurrence, it is reasonable to consider ILD as a differential diagnosis for non-specific pulmonary cases. The sample in the study from which the case vignettes were sourced^9^ was able to recognize ILD with a frequency appropriate for inferential statistical analysis. One potential explanation is that our study included only EPs compared to a blended sample of emergency and internal medicine physicians. Diagnoses of ILD are typically made in the outpatient pulmonary setting. These patients may present to the ED undifferentiated with non-specific respiratory symptoms that require admission and inpatient evaluation after appropriate stabilization. While ILD may be the etiology of a medical emergency, it is not an issue that must immediately be ruled out in the ED; accordingly, it may not be front of mind among EPs. Future research using this case may also consider adjustment such that identification of ILD is more obtainable.

An important aspect of our pilot study was using pre-existing cases without altering their content, recognizing that some pathologies may not be as apparent to EPs as they are to internists. We prioritized the PE case given its clinical relevance to a cohort of EPs, by presenting it first to all physicians. This further ensured that practice and attrition effects would spare the data related to the assessment of PE. Future studies that assess for the framing bias would benefit from creating test cases tailored toward medical pathologies with the participants’ specialty in mind rather than previously studied vignettes.

This investigation was a small, grant-funded, single-site pilot study. Our sample size was limited based on funding availability. A larger sample size might have strengthened existing findings or allowed investigation of those associations for which significance was not achieved, particularly the effect of time pressure upon diagnostic reasoning, which has previously been demonstrated among internal medicine residents.^10^ Our original goal was to test for an interaction effect between cognitive frame and time pressure, but our sample size was insufficient. The study population of EPs additionally limits generalizability to other groups of clinicians.

## CONCLUSION

Cognitive framing appears to influence both the likelihood of identifying a diagnosis and the order in which a diagnosis of interest is identified. This finding demonstrates an important source of diagnostic influence that clinicians must strive to mitigate. Future investigations should introduce a cognitive frame into a more realistic clinical situation via simulation or explore an alternative point of the care process, such as sign-out, while using cases tailored to the specialty of participants. Research into debiasing efforts is also critical to help diagnosticians avoid this cognitive pitfall.

## Supplementary Information







## Figures and Tables

**Figure:**
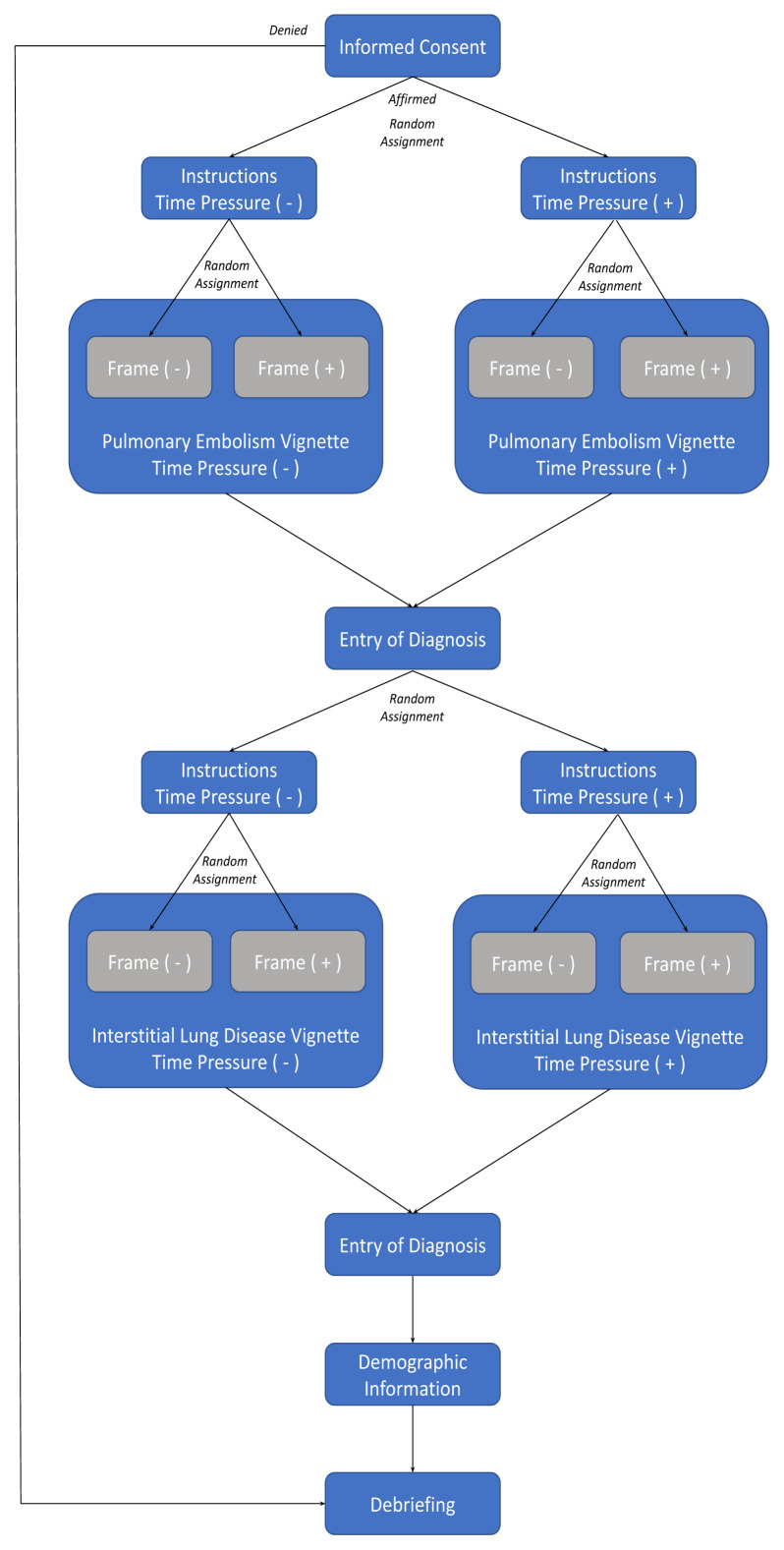
Participant process flow in a study of cognitive frame and time pressure as moderators of clinical reasoning.

**Table:** Main results in a study of cognitive frame and time pressure as moderators of clinical reasoning.

	Likelihood of identifying intended diagnosis		Likelihood of identifying intended diagnosis first among differential	
Condition	*P*	*n*	*P*	*n*
Application of cognitive frame	.01	39	.01	32
Application of time pressure	.09	39	.02	32
