# Supplementary figures and images for: Cognitive Frame and Time Pressure as Moderators Of Clinical Reasoning: A Case Control Study
Source: West J Emerg Med. 2025 Jul 11;26(4):1055–61. doi: 10.5811/westjem.24851 (PMC12342470; doi:10.5811/westjem.24851)

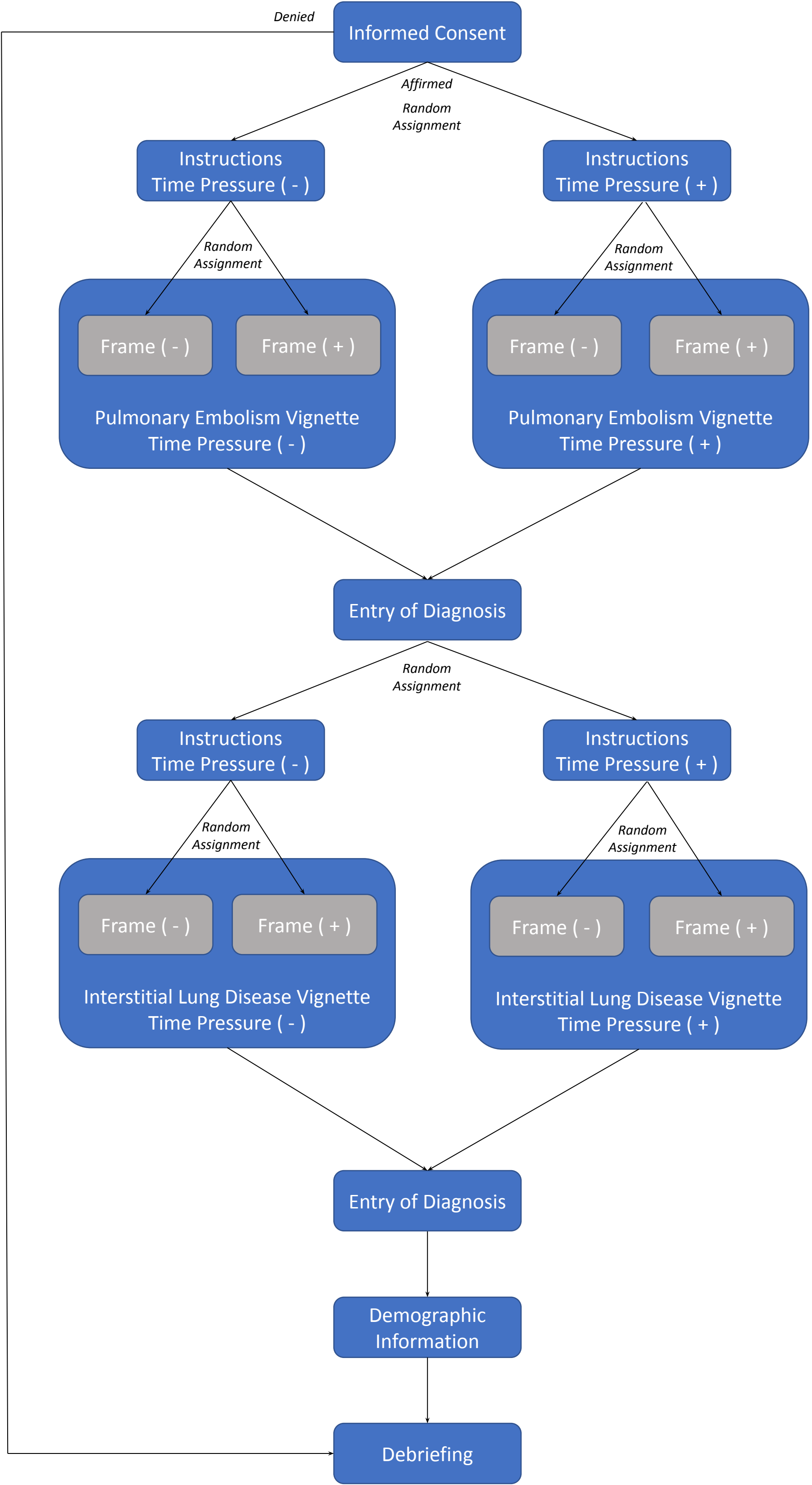

Supplement: Supplementary file 1 [file wjem-26-1055-g001.pdf]
