# Supplementary material for: Cognitive Frame and Time Pressure as Moderators Of Clinical Reasoning: A Case Control Study
Source: West J Emerg Med. 2025 Jul 11;26(4):1055–61. doi: 10.5811/westjem.24851 (PMC12342470; doi:10.5811/westjem.24851)
Supplement: Supplementary file 2 [file wjem-26-1055-s001.docx]

**Appendix 1 – Questionnaire: Pulmonary Embolism Case Vignettes**

*Framed to emphasize features consistent with PE:*

Gerald is a 65-year-old man with recent cholecystectomy presenting with shortness of breath and hemoptysis for 1 day in duration with associated tachycardia. He had gallbladder surgery 4 weeks ago. There has been no leg swelling since then. His shortness of breath came on gradually and is present at rest as well as increasing with exertion. His hemoptysis consists of some fresh blood mixed in with clear sputum, on several occasions yesterday. He denies any chest pain, dizziness, subjective fevers, or palpitations. He has had some upper respiratory tract infection-sounding symptoms in the past week with some cough and coryza. He has a past history of stable angina on aspirin and nitroglycerin, COPD on tiotropium bromide (Spiriva), and gallstones. Apart from a tachycardia of 110, his examination reveals a temperature of 37.8 degrees Celsius, saturations 96% on air, blood pressure of 150/90 mmHg, and some bibasilar crackles, with the remainder of the examination being normal. ECG is in sinus rhythm, with no other abnormalities. Full blood count and urea/electrolytes are all normal.

*Framed to emphasize features consistent with other diagnoses:*

Gerald is a 65-year-old man with a background of COPD presenting with a week of cough and coryzal symptoms and now several episodes of fresh hemoptysis mixed in with clear sputum in the last day. He has associated shortness of breath, both at rest and worse with exertion, that has come on gradually in the last day. He does not have any chest pain, dizziness, subjective fevers, palpitations, or leg swelling. His other medical history includes stable angina and gallstones, for which he has had laparoscopic cholecystectomy 1 month ago. He is on tiotropium bromide (Spiriva) for his COPD and also takes aspirin and nitroglycerin. Examination reveals low grade fever at 37.8 degrees Celsius, saturations of 96% on air, pulse 110, BP 150/90 mmHg, and bibasilar crackles. The remainder of the examination is normal. ECG shows sinus rhythm with no other abnormalities. Full blood count and urea/electrolytes are all normal.

*Adapted with permission from Popovich et al. (2019).*
