# Supplementary material for: Cognitive Frame and Time Pressure as Moderators Of Clinical Reasoning: A Case Control Study
Source: West J Emerg Med. 2025 Jul 11;26(4):1055–61. doi: 10.5811/westjem.24851 (PMC12342470; doi:10.5811/westjem.24851)
Supplement: Supplementary file 3 [file wjem-26-1055-s002.docx]

**Appendix 2 – Questionnaire: Interstitial Lung Disease Case Vignettes**

*Framed to emphasize features consistent with ILD:*

Mavis is an 80-year-old woman who has been on nitrofurantoin prophylaxis for recurrent UTIs who presents with a 1-year history of worsening shortness of breath, leaving her only able to walk 50 meters, and two weeks of suddenly worse shortness of breath, at rest, and cough. Her cough has been productive with white/yellow sputum, although she normally produces a fair amount of sputum daily due to her COPD. She has also been short of breath with lying flat and wakes up at night short of breath. She denies fevers, chest pain, palpitations, dizziness, or any other symptoms. Her background is recurrent UTIs being treated prophylactically with nitrofurantoin as already mentioned, atrial fibrillation, STEMI in 2007 with an ejection fraction at the time of 40%, angina with 100 meters, COPD with an FEV1 of 65% predicted in 2011, osteoarthritis of both knees, and GERD. Her medications are nitrofurantoin, aspirin, metoprolol, losartan, atorvastatin, sublingual nitroglycerin, warfarin, tiotropium, salmeterol, ipratropium, diclofenac, paracetamol, and omeprazole. She lives with her husband in their own house in an urban area. Her husband performs most activities of daily living for them these days. Examination reveals bibasilar crackles, saturations of 89% on air, up to 94% on 2L oxygen via nasal cannula and a respiratory rate of 25. Other observations are normal. There is also a bilateral wheeze listening to the chest, but equal and good air entry. The remainder of the examination is unremarkable and there is no other evidence of overload. ABG reveals a PaO2 of 75, with normal CO2 and other parameters. Bloods are normal except for a white cell count of 14. BNP is raised. ECG shows old Q waves in lateral leads. Chest X-ray is pending.

*Framed to emphasize features consistent with other diagnoses:*

Mavis is an 80-year-old woman presenting with shortness of breath. She has a history of atrial fibrillation, STEMI in 2007 with an ejection fraction at the time of 40%, angina with 100 meters, COPD with an FEV1 of 65% predicted in 2011, osteoarthritis in both knees, GERD, and recurrent UTIs. She has presented with a 2-week history of suddenly worse shortness of breath associated with nocturnal symptoms and cough productive of yellow/white sputum. She is short of breath at rest at the moment. This is on a background of 1 year of worsening shortness of breath leaving her only able to walk 50 meters. She is waking up at night short of breath and cannot tolerate lying flat. She produces a fair amount of sputum daily anyway due to her COPD. She denies chest pain, palpitations, dizziness, fevers, or any other symptoms. Her medications are aspirin, metoprolol, losartan, atorvastatin, sublingual nitroglycerin, warfarin, tiotropium, salmeterol, ipratropium, ipratropium, nitrofurantoin for UTI prophylaxis, diclofenac, paracetamol, and omeprazole. She lives with her husband in their own house in an urban area. Her husband performs most activities of daily living for them these days. Examination reveals oxygen saturations of 89% on air, up to 94% on 2L oxygen via nasal cannula, and a respiratory rate of 25, with other observations being normal. Listening to the chest, there is a widespread wheeze, good and equal air entry, and bibasilar crackles. The remainder of the examination is normal, with no other evidence of overload. Chest X- ray is pending. ECG shows old Q waves in lateral leads. BNP is elevated and the white cell count is 14, but other bloods are normal. An ABG shows normal CO2, with the only abnormality being a PaO2 of 75.

*Adapted with permission from Popovich et al. (2019).*
